# Nucleotide-Binding Oligomerization Domain 1/Toll-Like Receptor 4 Co-Engagement Promotes Non-Specific Immune Response Against K562 Cancer Cells

**DOI:** 10.3389/fphar.2022.920928

**Published:** 2022-07-22

**Authors:** Samo Guzelj, Žiga Jakopin

**Affiliations:** Faculty of Pharmacy, University of Ljubljana, Ljubljana, Slovenia

**Keywords:** NOD1 agonist, TLR4 agonist, LPS, synergy, cytolytic activity, PBMC, NK cells, K562

## Abstract

Nucleotide-binding oligomerization domain 1 (NOD1) receptor and Toll-like receptor 4 (TLR4) belong to the family of pattern recognition receptors. Interactions between these receptors profoundly shape the innate immune responses. We previously demonstrated that co-stimulation of peripheral blood mononuclear cells (PBMCs) with D-glutamyl-*meso*-diaminopimelic acid (iE-DAP)-based NOD1 agonists and lipopolysaccharide (LPS), a TLR4 agonist, synergistically increased the cytokine production. Herein, we postulate that stimulation of NOD1 alone or a combined stimulation of NOD1 and TLR4 could also strengthen PBMC-mediated cytotoxicity against cancer cells. Initially, an in-house library of iE-DAP analogs was screened for NOD1 agonist activity to establish their potency in HEK-Blue NOD1 cells. Next, we showed that our most potent NOD1 agonist SZZ-38 markedly enhanced the LPS-induced cytokine secretion from PBMCs, in addition to PBMC- and natural killer (NK) cell-mediated killing of K562 cancer cells. Activation marker analysis revealed that the frequencies of CD69^+^, CD107a^+^, and IFN-γ^+^ NK cells are significantly upregulated following NOD1/TLR4 co-stimulation. Of note, SZZ-38 also enhanced the IFN-γ-induced PBMC cytotoxicity. Overall, our findings provide further insight into how co-engagement of two pathways boosts the non-specific immune response and attest to the importance of such interplay between NOD1 and TLR4.

## Introduction

Nucleotide-binding oligomerization domain protein 1 (NOD1) is an innate immune receptor, expressed extensively in numerous cell types, which senses portions of bacterial peptidoglycan (Inohara et al., 2001; Philpott et al., 2014) featuring the D-glutamyl-*meso*-diaminopimelic acid (iE-DAP) motif (Girardin et al., 2003a; Girardin et al., 2003b; Girardin et al., 2005; Uehara et al., 2005). It is involved in the generation of innate immune responses by engaging the nuclear factor κB (NF-κB) and mitogen-associated protein kinase signaling pathways (Inohara et al., 1999; Chamaillard et al., 2003; Strober et al., 2006), and plays a minor role in several other innate immunity-related mechanisms (Jakopin, 2014), such as autophagy (Travassos et al., 2010) and apoptosis (Inohara and Nuñez, 2003).

The NOD1 activating capacity of iE-DAP has been successfully augmented, mostly by appending lipophilic alkyl/acyl chains to its D-Glu portion (Hasegawa et al., 2007; Agnihotri et al., 2011; Fujimoto and Fukase, 2011). According to these findings, we prepared iE-DAP derivatives harboring the lauroyl or didodecyl substituents attached to the amino group of the D-Glu residue. Importantly, those compounds also featured a double bond functionality installed into the *meso*-DAP side-chain, which gave rise to potent NOD1 agonists (Jakopin et al., 2013). *Meso*-DAP side-chain, however, is long and flexible, hence capable of assuming diverse spatial conformations, not all of which are optimal for binding NOD1. Rigidization in an optimal conformation thus improved the NOD1 agonistic activity of the parent compound. In our ensuing exploration of the chemical space around the *meso*-DAP portion, we incorporated isoxazoline- and pyridine-based constrained *meso*-DAP mimetics into the structure of iE-DAP, which abrogated their NOD1 activating capacity (Guzelj and Jakopin, 2020).

A comprehensive study of biological activities of one of the earliest identified NOD1 agonists, the tetrapeptide FK-156 and the tripeptide FK-565, revealed significant therapeutic potential; in addition to strengthening the innate and adaptive immune responses, in turn increasing the host defence against various pathogens (Mine et al., 1983a; Mine et al., 1983b; Yokota et al., 1983), they also exhibited pronounced antitumor activity (da Silva Correia et al., 2006; da Silva Correia et al., 2007; Watanabe et al., 1985; Nabergoj et al., 2019). In spite of the fact that the role of the NOD1 protein in tumorigenesis is ambiguous (Zhan et al., 2016; Jiang et al., 2020; Rommereim et al., 2020), its agonists have shown the ability to enhance the non-specific antitumor activity of immune effector cells often present in the tumor microenvironment, in particular monocytes and macrophages but also natural killer (NK) cells, and consequently facilitating their engagement with diverse cancer cell lines, including Tu167 squamous cell carcinoma, P815 mastocytoma, K562 chronic myelogenous leukemia, Raji lymphoblastoid lymphoma, and A375 melanoma (Schultz and Altom, 1986; Wang et al., 1989a; Wang et al., 1989b; Qiu et al., 2010). On top of that, NOD1 agonists have the capacity to strengthen the antigen-specific variant of antitumor immunity either as vaccine adjuvants (Jacoberger-Foissac et al., 2019; Zhao et al., 2019; Jacoberger-Foissac et al., 2020) or by enhancing dendritic cell (DC) cross-priming (Asano et al., 2010).

iE-DAP and its natural/synthetic derivatives are moderate stimulants of immune cells, in terms of the induced pro-inflammatory cytokine secretion. This effect, however, can be potentiated if combined with lipopolysaccharide (LPS), a Toll-like receptor 4 (TLR4) agonist, and many other TLR agonists (Tada et al., 2005; Van Heel et al., 2005; Masumoto et al., 2006; Van Heel et al., 2006). Such interplay profoundly shapes the innate immune responses as the synergistic responses were seen across multiple receptors and a wide range of secreted cytokines. In this manner, NOD1 agonists offer an alternative pathway to hosts refractory to TLR signals (Kim et al., 2008). In addition, NOD1 agonists have been reported to potentiate the biological activities of endogenous proteins/molecules, such as interferon-γ (IFN-γ) (Sone et al., 1988; Inamura et al., 1989), monocyte-activating factor (Schultz and Altom, 1986), anti-CD3 (Zhan et al., 2016), interleukin-2 (IL-2) (Wang et al., 1989a), as well as certain chemotherapeutics (Ma et al., 2020) and antibiotics (Yokota et al., 1983). The immunostimulatory potential of NOD1 agonists has been investigated in human primary peripheral blood mononuclear cells (PBMCs), which constitute a heterogeneous population of immune cells, all of which express functional NOD1 protein. Different NOD1 agonists (in the 100 nM–1 µM range) elicited only modest pro-inflammatory cytokine secretion from PBMCs by themselves but synergistically increased the LPS-induced responses (Van Heel et al., 2005; Van Heel et al., 2006). Similarly, we demonstrated that co-stimulation of PBMCs with NOD1 agonists and LPS brought about a substantial increase in cytokine production (Jakopin et al., 2013). It is worth noting that NOD1/TLR4 synergy extends across different cell types and has also been observed in macrophages (Budikhina et al., 2021), bone-marrow derived DCs (BMDCs) (Masumoto et al., 2006; Xu and Daicoli, 2010), DCs (Tada et al., 2005), invariant natural killer T cells (Selvanantham et al., 2013) and mesothelial cells (Park et al., 2007).

**FIGURE 1:**
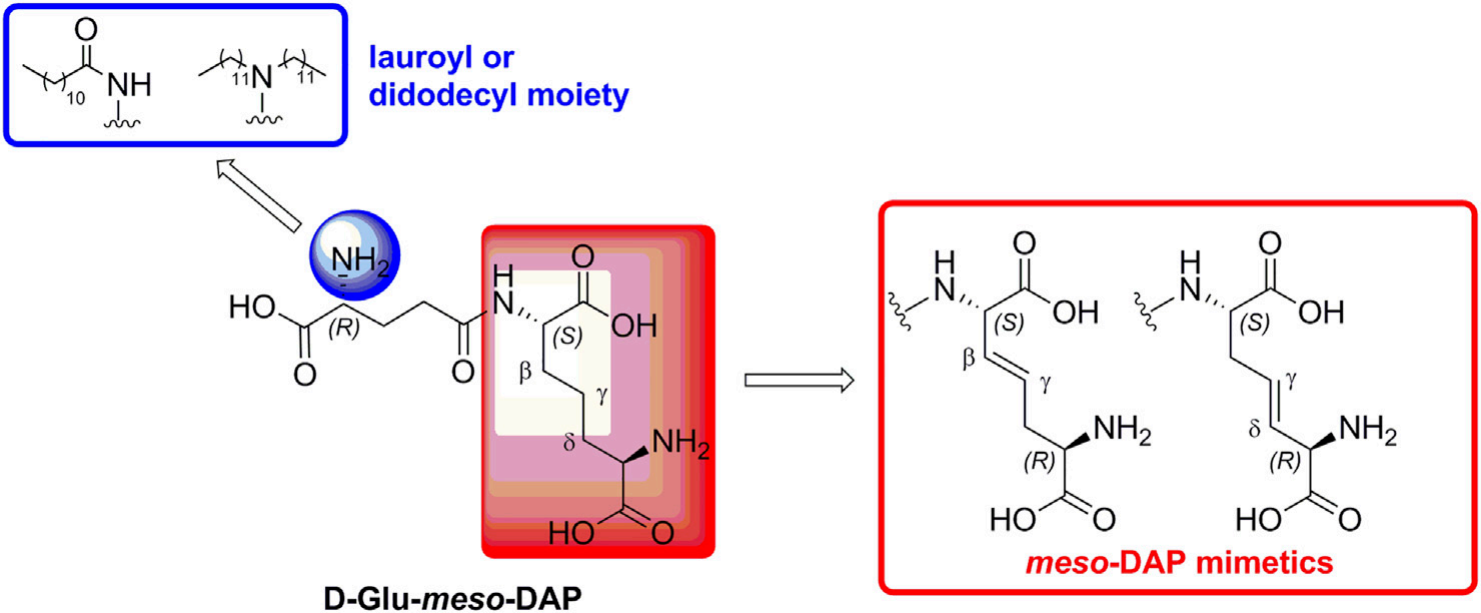
Structural modifications of the parent D-Glu-*meso*-DAP (iE-DAP) resulting in conformationally constrained analogs.

In our previously prepared small library of four constrained iE-DAP analogs, a double bond functionality was installed into the side chain of the *meso*-DAP residue at two different sites, βγ and γδ, while lauroyl or didodecyl moieties were attached to the D-Glu amino group (see Figure 1). At the time, Ramos-Blue cell line was used as a model for studying their NOD1 agonistic activity *via* their ability to induce activation of NF-κB. The test, however, was carried out only at a single and, notably, rather high concentration of 10 µM. Consequently, the obtained results which displayed a maximum NOD1 activation of all four compounds did not provide a good basis to discern between their NOD1 agonistic activities and establish a basic structure-activity relationship. The initial aim of this study was therefore to ascertain the effect of the following structural changes (see Figure 1): 1) replacement of the *meso*-DAP with its constrained mimetics (Figure 1, blue) and 2) installment of lauroyl and dodecyl functionalities at the D-Glu amine (Figure 1, red). The HEK-Blue NOD1 cell line served as a model for investigating the ability of these compounds to activate NF-κB through NOD1 activation. The capacity of the most potent NOD1 agonist to elicit cytokine production by itself or in synergy with LPS was established in PBMCs. Next, a flow cytometry-based PBMC cytotoxicity assay was utilized to delineate whether either stimulation of NOD1 alone or a combined stimulation of NOD1 and TLR4 can strengthen the cytolytic activity of PBMCs and NK cells against K562 cancer cells. Moreover, a functional flow cytometric panel was employed to determine the effects of NOD1/TLR4 co-engagement on NK cell activation, degranulation, and production of IFN-γ. Finally, the effect of dual stimulation with NOD1 agonist and IFN-γ on the cytolytic activity of PBMCs has been elucidated.

## Materials and Methods

### General Information

C12-iE-DAP (a synthetic NOD1 agonist) was obtained from InvivoGen, Inc., (San Diego/CA, United States); LPS (from *Escherichia coli* serotype 0127:B8) was from Sigma-Aldrich, Inc. (St. Louis/MO, United States). NOD1 antagonist Nodinitib-1 (Correa et al., 2011) and TLR4 antagonist TLR4-IN-C34 (Neal et al., 2013) were from MedChemExpress (NJ, United States). IL-2 and IFN-γ were from PeproTech (London, United Kingdom). The conformationally constrained NOD1 agonists (SZZ-38, SZZ-39, SZZ-40, and SZZ-41) were synthesized as described (Jakopin et al., 2013). The HPLC purity of all pharmacologically investigated compounds was >95%. Stock solutions of chemicals were prepared in DMSO before use and the final concentration of DMSO never exceeded 0.1%.

### HEK-Blue Nucleotide-Binding Oligomerization Domain 1 Cells

HEK-Blue NOD1 cells (Invivogen, San Diego/CA, United States) were cultured in accordance with the manufacturer’s instructions in DMEM medium (Sigma-Aldrich, St. Louis/MO, United States) supplemented with 10% heat-inactivated FBS (Gibco, ThermoFisher Scientific, Waltham/MA, United States), 2 mM L-glutamine (Sigma-Aldrich, St. Louis/MO, United States), 100 U/mL penicillin (Sigma-Aldrich), 100 µg/mL streptomycin (Sigma-Aldrich, St. Louis/MO, United States), and 100 µg/mL Normocin (Invivogen, San Diego, CA, United States) for 2 passages. All subsequent passages were cultured in the medium additionally supplemented with 30 µg/mL Blasticidin (Invivogen, San Diego/CA, United States) and 100 µg/mL Zeocin (Invivogen, San Diego/CA, United States). The cells were incubated in a humidified atmosphere at 37°C and 5% CO_2_.

### Peripheral Blood Mononuclear Cells and Natural Killer Cells

Human PBMCs from healthy and consenting donors were isolated from heparinized blood by density gradient centrifugation with Ficoll-Paque (Pharmacia, Sweden). NK cells were isolated from heparinized blood using the RosetteSep Human NK Cell Enrichment Cocktail (StemCell Technologies, Vancouver, Canada) in accordance with the manufacturer’s instructions. The purity of NK cells was evaluated with an-ti-CD3 APC/Fire 750 and anti-CD56 PE antibodies by flow cytometry with >90% shown to be CD3^−^ CD56^+^.

The isolated cells were resuspended in RPMI 1640 medium (Sigma-Aldrich, St. Louis/MO, United States) supplemented with 10% heat-inactivated FBS (Gibco, ThermoFisher Scientific, Waltham/MA, United States), 2 mM L-glutamine (Sigma-Aldrich, St. Louis/MO, United States), 100 U/mL penicillin (Sigma-Aldrich, St. Louis/MO, United States), and 100 µg/mL streptomycin (Sigma-Aldrich, St. Louis/MO, United States) and used for assays.

### K562 Cells

K562 is a chronic myelogenous leukemia cell line (ATCC, Manassas, VA, United States), which was cultured in RPMI 1640 medium (Sigma-Aldrich, St. Louis/MO, United States) supplemented with 10% heat-inactivated FBS (Gibco, ThermoFisher Scientific, Waltham/MA, United States), 2 mM L-glutamine (Sigma-Aldrich, St. Louis/MO, United States), 100 U/mL penicillin (Sigma-Aldrich, St. Louis/MO, United States), and 100 µg/mL streptomycin (Sigma-Aldrich, St. Louis/MO, United States).

### Cytotoxicity Assay

HEK-Blue NOD1 cells (4 × 10^5^ cells/mL; 40,000/well) were treated with the appropriate amounts of compounds or with the corresponding vehicle (control cells), then seeded in duplicate in 96-well plates. After 20 h, the metabolic activity was assessed using the CellTiter 96^®^ Aqueous One Solution Cell Proliferation Assay (Promega, Madison/WI, United States), in accordance with the manufacturer’s instructions. The results are expressed as means of duplicates ± SEM of two independent experiments.

### Measurement of Nuclear Factor κB Transcriptional Activity (Quanti-Blue Assay)

HEK-Blue NOD1 cells were assayed for changes in the NF-κB transcriptional activity upon incubation (2.5 × 10^5^ cells/mL; 25,000/well) with compounds (1 µM for the fixed concentration assay; eight different concentrations ranging from 0.1 nM to 10 µM for the determination of EC_50_ values) or with the corresponding vehicle (0.1% DMSO) for 20 h. The secreted embryonic alkaline phosphatase (SEAP) activity was determined in the supernatant in accordance with the manufacturer’s instructions. Absorbance was measured on a BioTek Synergy microplate reader (BioTek Instruments, Inc., Winooski, United States) at 640 nm. The results are expressed either as the means ± SEM of duplicates of two independent experiments (single concentration) or means ± S.E.M. of four independent experiments (EC_50_ values).

### Cytokine Release From Peripheral Blood Mononuclear Cells

PBMCs were seeded (150,000 cells/well) in 96-well plates in 150 µl growth medium and treated with SZZ-38 (1 µM), LPS (2 ng/mL), both, or the corresponding vehicle (0.1% DMSO). In some wells, PBMCs were pre-treated for 1 h with the NOD1 antagonist Nodinitib-1 (10 µM), TLR4 antagonist TLR4-IN-C34 (100 µM), or both, before the addition of SZZ-38 and/or LPS. Cell-free supernatants were collected after 18 h of incubation (37°C, 5% CO_2_) and stored at −80°C until tested. Cytokine concentrations were determined with the LEGENDplex Human Th Cytokine Panel (Biolegend, San Diego, CA, United States) on an Attune NxT flow cytometer (Thermo Fisher Scientific, Waltham, MA, United States) in accordance with manufacturer’s instructions. Standard curves were generated using recombinant cytokines contained in the kit. The data were analysed using the LEGENDplex Data Analysis Software Suite (BioLegend).

### Peripheral Blood Mononuclear Cells-K562 and Natural Killer Cells-K562 Cytotoxicity Assays

The PBMC and NK cell cytotoxicity assays using K562 cells were carried out as described previously with a few modifications (Kandarian et al., 2017). PBMCs (4 × 10^5^ cells/well) or isolated NK cells (5 × 10^4^ cells/well) were seeded on 96-well plates and treated with com-pounds (10 nM–10 µM) or vehicle (0.1% DMSO) for 18 h. In synergy studies, cells were additionally stimulated with LPS at a final concentration of 10 ng/mL–1 µg/mL, or IFN-γ at a final concentration of 10 ng/mL–100 ng/mL as indicated in the figure legends. In experiments with NOD1 and TLR4 antagonists, PBMCs were pre-treated with a NOD1 antagonist Nodinitib-1 (10 µM), TLR4 antagonist TLR4-IN-C34 (100 µM), or both for 1 h, before the addition of SZZ-38 and LPS. IL-2 (200 IU/mL) was used as the positive control.

K562 cells were stained with CFSE (Invitrogen, Carlsbad/CA, United States), washed twice with complete medium, and added (1 × 10^4^ cells/well) to pre-treated effector cells for a final effector cell to target tumor cell ratio of 40:1 in the case of PBMCs, and 5:1 in the case of isolated NK cells. After a 4 h co-incubation period (37°C, 5% CO_2_), cells were stained with Sytox Blue dead cell stain (Invitrogen, Carlsbad/CA, United States) and analyzed with an Attune NxT flow cytometer (Thermo Fisher Scientific, Waltham/MA, United States) and FlowJo software (Tree Star, Inc., Ashland/OR, United States). Cells that were positive for both CFSE and Sytox blue were assessed as dead K562 cells. PBMCs, NK cells, and CFSE-labeled K562 cells alone were also treated with compounds at the same concentrations and stained with Sytox blue to assess direct cytotoxicity of compounds towards PBMCs, NK cells, and K562 cells, respectively. The experiments were run in duplicates and repeated in three independent biological replicates.

### Natural Killer Cell Degranulation, Activation, and Production of Interferon-γ

PBMCs were seeded (5 × 10^5^ cells/well) on 96-well plates and treated with SZZ-38 (1 µM), LPS (1 µg/mL), both, or the corresponding vehicle (0.1% DMSO) for 24 h. Anti-CD107a FITC antibody and monensin (Biolegend, San Diego, CA, United States) were added to all wells for the last 4 h of incubation. Cells were washed and stained with Live/Dead Fixable Aqua Dead Cell Stain (Invitrogen, Carlsbad, CA, United States). After further washing, Fc receptors were blocked with Human TruStain FcX (Biolegend) and cells were stained for extracellular markers using anti-CD3 APC/Fire 750, anti-CD56 PE, and anti-CD69 PerCP-Cy5.5 antibodies (Biolegend). Intracellular staining was performed with anti-IFN-γ APC antibody (Biolegend) after fixation and permeabilization using the Cyto-Fast Fix/Perm Buffer Set (Biolegend). Samples were analyzed using an Attune NxT flow cytometer (Thermo Fisher Scientific, Waltham, MA, United States) and FlowJo software (Tree Star, Inc., Ashland, OR, United States). Following exclusion of debris and dead cells, CD3^−^ CD56^+^ NK cells were evaluated for expression of CD107a, CD69, and IFN-γ.

### Data Analysis and Statistics

All the experiments were performed at least two times, with average values expressed as means ± standard error of mean (SEM). Statistical analyses were performed using GraphPad Prism 9 (La Jolla/CA, United States). Statistical significance was determined by one- or two-way ANOVA followed with the Dunnet’s multiple comparisons test. Differences were considered significant (*) for *p* < 0.05, highly significant (**) for *p* < 0.01, and extremely significant (***) for *p* < 0.001.

## Results and Discussion

### Effect of Nucleotide-Binding Oligomerization Domain 1 Agonists on Cell Viability

**FIGURE 2:**
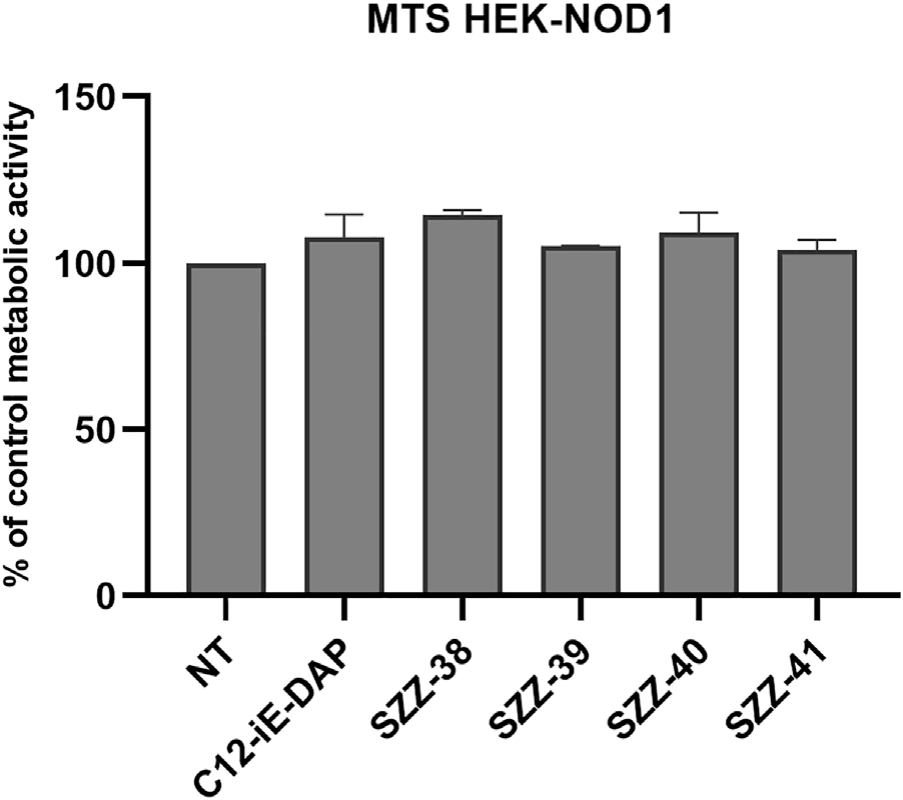
Proliferation rates of NOD1-specific HEK-Blue cells following 20 h incubation with C12-iE-DAP (1 µM) and constrained iE-DAP derivatives SZZ-38, SZZ-39, SZZ-40, and SZZ-41 (all at 1 µM). The rates, expressed as means of duplicates ± SEM of two independent experiments, are shown relative to that of the control (NT).

The MTS metabolic activity assay was utilized to ascertain whether the tested compounds affect cell viability by measuring the proliferation rates of NOD1-specific HEK-Blue cells in the presence of the reference NOD1 agonist C12-iE-DAP (Agnihotri et al., 2011; Tukhvatulin et al., 2011) and the constrained iE-DAP analogs. Cells were treated for 20 h with the compound of interest (1 µM). The comparison of the measured metabolic activities with that of the untreated control highlighted a complete absence of cytotoxicity for HEK-Blue NOD1 cells, since none of the residual metabolic activities fell below 90% (Figure 2).

### Determination of Nucleotide-Binding Oligomerization Domain 1 Agonist Activities

The conformationally restricted NOD1 agonists and C12-iE-DAP were then examined for their NOD1-activating capacity using the commercially available NOD1-specific HEK-Blue assay. Besides expressing the hNOD1 gene, these cells also express an NF-κB-inducible secreted embryonic alkaline phosphatase (SEAP) reporter gene. Upon binding of a NOD1 agonist to its cognate receptor, a signaling cascade leading to the activation of NF-κB is triggered, in turn resulting in the production of SEAP. HEK-Blue NOD1 cells were incubated for 20 h with C12-iE-DAP (1 µM) and the constrained NOD1 agonists SZZ-38, SZZ-39, SZZ-40, and SZZ-41 (1 µM); the results are collected in Table 1. As expected, all tested compounds and the positive control, C12-iE-DAP, substantially augmented the NF-κB transcriptional activity relative to that of untreated cells. The compounds were further investigated for their dose dependence and EC_50_ values (Table 1).

**TABLE 1:** NOD1 agonistic activities of conformationally constrained iE-DAP derivatives.

Compound	Structure	NOD1 activity at 1 µM^a^	NOD1 EC_50_ ^b^
C12-iE-DAP	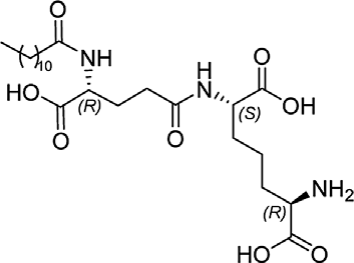	7.20 ± 0.56	170 ± 37.6 nM
SZZ-38	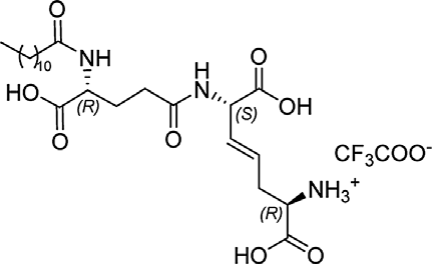	8.69 ± 0.66	46 ± 5.1 nM
SZZ-39	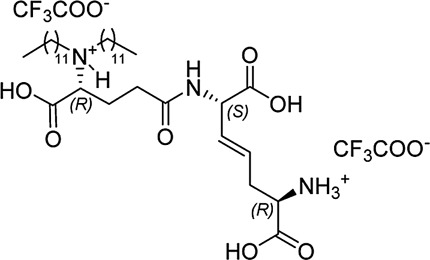	7.31 ± 0.93	162 ± 11.6 nM
SZZ-40	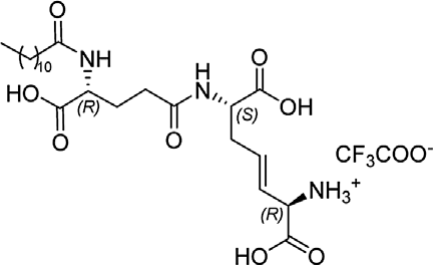	7.36 ± 0.73	686 ± 12.5 nM
SZZ-41	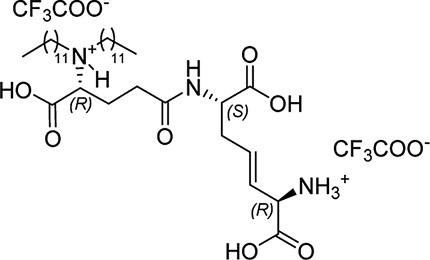	6.90 ± 0.58	127 ± 16.8 nM

a SEAP activities were measured in NOD1-specific HEK-Blue cell supernatants after incubation for 20 h with compounds of interest (1 μM) or C12-iE-DAP (1 μM). The data are shown relative to that of the negative control (0.1% DMSO) and expressed as means ± SEM of duplicates of two independent experiments.

b EC_50_ values were measured in NOD1-specific HEK-Blue cell supernatants after incubation for 20 h; data are shown as means ± SEM of four independent experiments with 8 data points ranging from 0.1 nM to 10 µM.

Compound SZZ-38 that features N-lauroyl-D-Glu moiety in its structure and a double bond positioned at the βγ site was the best performing NOD1 agonist of the series (EC_50_, 46 ± 7.2 nM), exhibiting an almost 4-fold boost in activity compared to that of its flexible counterpart C12-iE-DAP (EC_50_, 170 ± 53.2 nM). Conversely, its regioisomer SZZ-40 carrying a γδ positioned double bond in the *meso*-DAP side-chain exhibited a substantial drop in NOD1 agonistic activity (EC_50_, 686 ± 17.7 nM). Interestingly, their close structural analogs SZZ-39 and SZZ-41, in which the D-Glu amine functionalities are decorated with didodecyl moieties, disparately modulated the NOD1 agonistic activity. In case of compound SZZ-39 (EC_50_, 162 ± 16.4 nM), a slight decrease in NOD1 activating capacity has been indicated compared to its N-lauroyl counterpart SZZ-38, while with compound SZZ-41 (EC_50_, 127 ± 23.7 nM), the effect was reversed, thus more than a 5-fold increase in potency has been achieved compared to the lauroyl-featuring compound SZZ-40. Importantly, obtained results show that guided constraining of the *meso*-DAP side-chain moiety can fine-tune the ability of compounds to activate NOD1.

### Effect of Nucleotide-Binding Oligomerization Domain 1 Agonists on Cytokine Secretion From Peripheral Blood Mononuclear Cells

**FIGURE 3:**
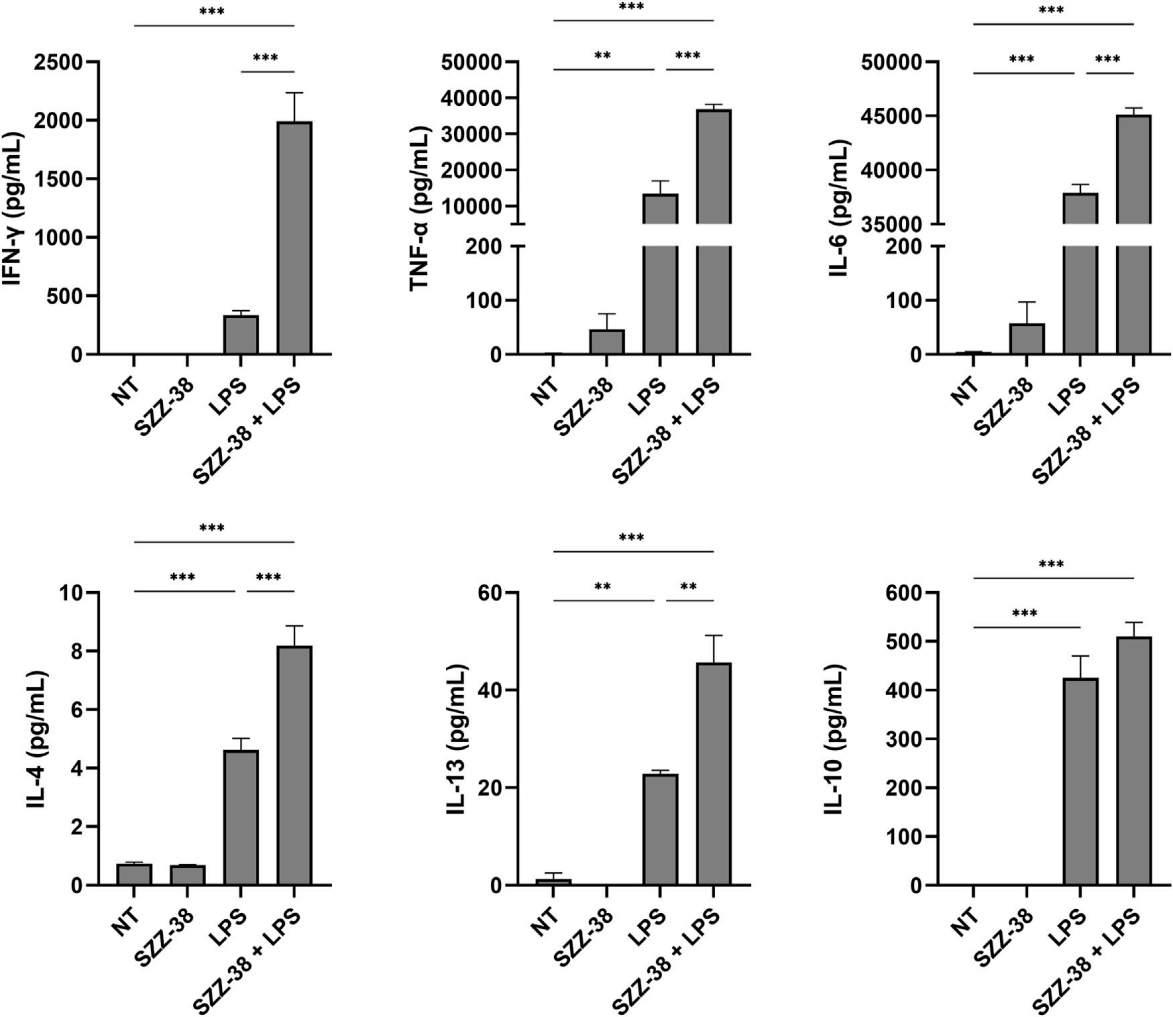
Synergistic effect of SZZ-38 and LPS on the induction of cytokine production from PBMCs. Cytokine concentrations were measured after 18 h stimulation with SZZ-38 (1 µM), LPS (2 ng/mL), both, or the corresponding vehicle (0.1% DMSO; NT). Data are expressed as means ± SEM of three independent experiments. Statistical significance was determined by one-way ANOVA followed by Bonferroni’s multiple comparisons test; ***, *p* < 0.001.

NOD1 agonists have been shown on several occasions to elicit modest pro-inflammatory cytokine production by themselves and have a marked synergistic effect on the production of TLR-mediated inflammatory cytokines *in vitro* (Tada et al., 2005; Van Heel et al., 2005; Masumoto et al., 2006; Van Heel et al., 2006; Jakopin et al., 2013). We therefore interrogated whether stimulation of NOD1 or a combined NOD1/TLR4 stimulation has a similar immunostimulatory effect in PBMCs. The capacity of the most potent NOD1 agonist SZZ-38 to induce cytokine production by itself or in synergy with LPS was assessed in human PBMCs. These were treated with SZZ-38 (1 µM), LPS (2 ng/mL), or a combination of SZZ-38 and LPS. The cytokine release was measured after 18 h using the LEGENDplex Human Th Cytokine Panel (Figure 3). SZZ-38 by itself induced a modest secretion of pro-inflammatory cytokines TNF-α and IL-6, while the release of other measured cytokines was not affected. SZZ-38 was then evaluated for its effect on cytokine release from LPS-stimulated PBMCs. Enhanced TLR4-mediated responses have been demonstrated at the level of multiple cytokines indicative of Th1 as well as Th2 and Treg responses. Of note, a strong synergy on IFN-γ production was observed. Similarly, the level of TNF-α was also significantly increased by a combination of NOD1/TLR4 agonists, while the release of IL-6 was only modestly augmented. Moreover, the synergistic effect of dual NOD1/TLR4 stimulation on the production of Th2 cytokines, IL-4 and IL-13, and the regulatory Th cytokine IL-10 was also seen but to a lesser extent.

The obtained results are in good agreement with those obtained by Jakopin et al. (2013) who reported synergistic responses of NOD1/TLR4 agonist combinations with IL-6, IL-10 and TNF-α release, while stimulation by NOD1 agonist alone brought about only modest increases in the levels of those cytokines (Jakopin et al., 2013). Moreover, Van Heel et al. (2006) also highlighted the capacity of NOD1 agonists to induce minor production of TNF-α and IL-8 from PBMCs (Van Heel et al., 2006). Importantly, NOD1 agonists acted in synergy with LPS to stimulate the release of both pro- and anti-inflammatory cytokines also from specific myeloid cell populations, including monocytes and DCs (Fritz et al., 2005).

The specificity of the observed synergistic effect on the cytokine release PBMCs was also examined. PBMCs were pre-treated with a reference NOD1 antagonist (Correa et al., 2011), TLR4 antagonist (Neal et al., 2013) or both antagonists prior to stimulation with SZZ-38 (1 µM), LPS (2 ng/mL), or a combination of SZZ-38 and LPS; corresponding vehicle (0.1% DMSO) was used as the negative control. In each case, the cytokine release was evaluated and compared to those of the controls not being subjected to antagonist(s) pre-treatment. The significant decreases in cytokine secretion observed in case one or both antagonists were used, strongly suggest that the augmented release of several cytokines induced by SZZ-38, LPS and their combination is indeed a consequence of their NOD1 and/or TLR4 activation (Supplementary Figure S1).

### Effect of Nucleotide-Binding Oligomerization Domain 1 Agonists on Peripheral Blood Mononuclear Cells-Mediated K562 Cell Killing

In order to delineate whether stimulation of NOD1 can boost the non-specific antitumor immunity, a well-established cytotoxicity assay has been used, based on the co-incubation of PBMCs with fluorescently labelled K562 cancer cells (Kandarian et al., 2017). Among the PBMC subfractions, NK cells in particular, but also monocytes, are heavily engaged in the generation of antitumor immune response. NK cells play a central role in the destruction of cancer cells *via* both direct cytolytic activity and release of cytokines, which in turn reinforces the recruitment and activation of other immune cells (Qiu et al., 2010). Similarly, monocytes can exert non-specific cytolytic activity against cancer cells (Wang et al., 1989a), but for the most part serve as accessory immune cells. We have demonstrated a significant amplification of IFN-γ production elicited by combined NOD1/TLR4 stimulation, which likely plays an important role in the PBMC-mediated cytotoxicity. For these reasons, the whole PBMC population was first utilized as effector cells in favor of isolated NK cells, thus recapitulating more realistic conditions. Specifically, we investigated if and how our NOD1 agonists modulate the PBMC cytolytic activity versus the target chronic myelogenous leukemia K562 cells as they are considered a “classic” NK cell target due to the lack of markers required for NK cell inhibition (Khanna et al., 2021).

**FIGURE 4:**
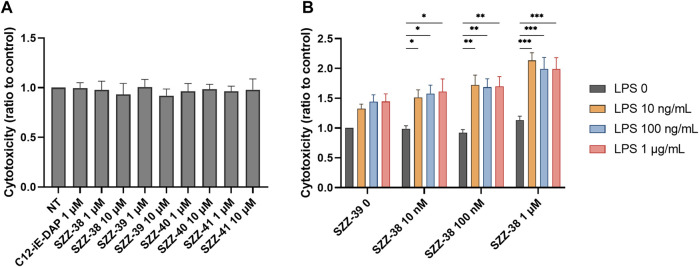
Effect of NOD1 agonists and LPS on the cytolytic activity of PBMCs against K562 cells. **(A)** PBMCs were treated for 18 h with C12-iE-DAP (1 µM) or NOD1 agonists (1 and 10 µM), before the addition of K562 cells. Cytotoxicity was determined after 4 h of co-incubation. **(B)** The concentration-dependent effect of NOD1/TLR4 agonist combinations on the induction of PBMC cytotoxicity. Data are shown relative to the negative control (0.1 % DMSO) and expressed as means ± SEM of three independent experiments. Statistical significance was determined by two-way ANOVA followed by Dunnets multiple comparisons test; *, p < 0.05, **, p < 0.01, ***, p < 0.001.

Firstly, PBMCs were treated for 18 h with compounds SZZ-38, SZZ-39, SZZ-40 and SZZ-41 (1 and 10 µM) and C12-iE-DAP (1 µM) as the reference NOD1 agonist before the addition of K562 at a 40:1 effector to target (E/T) cell ratio. The cytotoxicity measured after 4 h showed that none of the compounds amplified the cytolytic capacity of PBMCs at either concentration (Figure 4A). The obtained results are in agreement with those obtained by Qiu et al. (2010) who showed that iE-DAP was not able to augment the NK cell cytotoxicity against K562. They did, however, observe a cytotoxic effect towards the squamous cell carcinoma of head and neck cell line Tu167 (Qiu et al., 2010). Conversely, Yi-Li et al. demonstrated that FK-565 was able to increase markedly the NK cell cytotoxicity against K562 and Raji lymphoblastoid lymphoma (Wang et al., 1989b). Granted, FK-565 is a much more potent NOD1 agonist than iE-DAP.

Moreover, a combined NOD1/TLR4 stimulation has previously been shown to potentiate the antimicrobial response of NK cells *via* engagement of BMDCs (Selvanantham et al., 2013) as well as the antigen-specific antitumor immunity of BMDCs against B16-OVA melanoma (Xu and Daicoli, 2010). In order to elucidate whether a simultaneous engagement of NOD1 and TLR4 can also boost the non-specific arm of antitumor immunity, we turned our attention towards combined stimulations of PBMCs employing different concentrations of our most potent NOD1 agonist SZZ-38 (at 10 nM, 100 nM and 1 µM), alone or in combination with LPS (at 10, 100, and 1,000 ng/mL); again, K562 were used as target cells at a 40:1 E/T cell ratio (Figure 4B). In parallel, we also treated PBMCs and cancer cells separately to exclude the possibility of direct cytotoxicity of NOD1 agonist and/or NOD1/TLR4 agonist combinations towards PBMCs or K562 cells. None of the tested compounds caused any changes in the percentage of dead cells (data not shown), thereby confirming that the enhanced activity can solely be attributed to the stimulation of PBMC cytolytic activity. As expected, SZZ-38 alone (at 10 nM, 100 nM and 1 µM) was unable to enhance the cytolytic activity of PBMCs, while LPS as the positive control boosted the cytotoxicity of PBMCs against K562 at all tested concentrations. The augmented cytotoxicity was most pronounced at LPS concentrations of 100 nM (1.44-fold) and 1 µM (1.45-fold). Of note, the level of synergistic effect on PBMC cytotoxic capacity has clearly been shown to depend on the utilized concentrations of both agonists. A maximum level of synergy was achieved by a combination of 1 µM SZZ-38 and 10 ng/mLof LPS (2.13-fold), while this level declined slightly with increasing LPS concentrations, 100 ng/mL (1.99-fold) and 1 µg/mL (1.99-fold). In case of lower concentrations of SZZ-38 used (10 and 100 nM), the synergy, albeit significant throughout the whole range of LPS combinations, did not reach its full potential, which reflected in a lower cytotoxic capacity of PBMCs (ranging from 1.51- to 1.72-fold). Similar concentration-dependent phenomenon has also been reported for the induced cytokine secretion from PBMCs; most pronounced synergistic effect has been elicited with a combination of a NOD1 agonist at 100 nM and LPS at 1 ng/mL (Van Heel et al., 2006).

**FIGURE 5:**
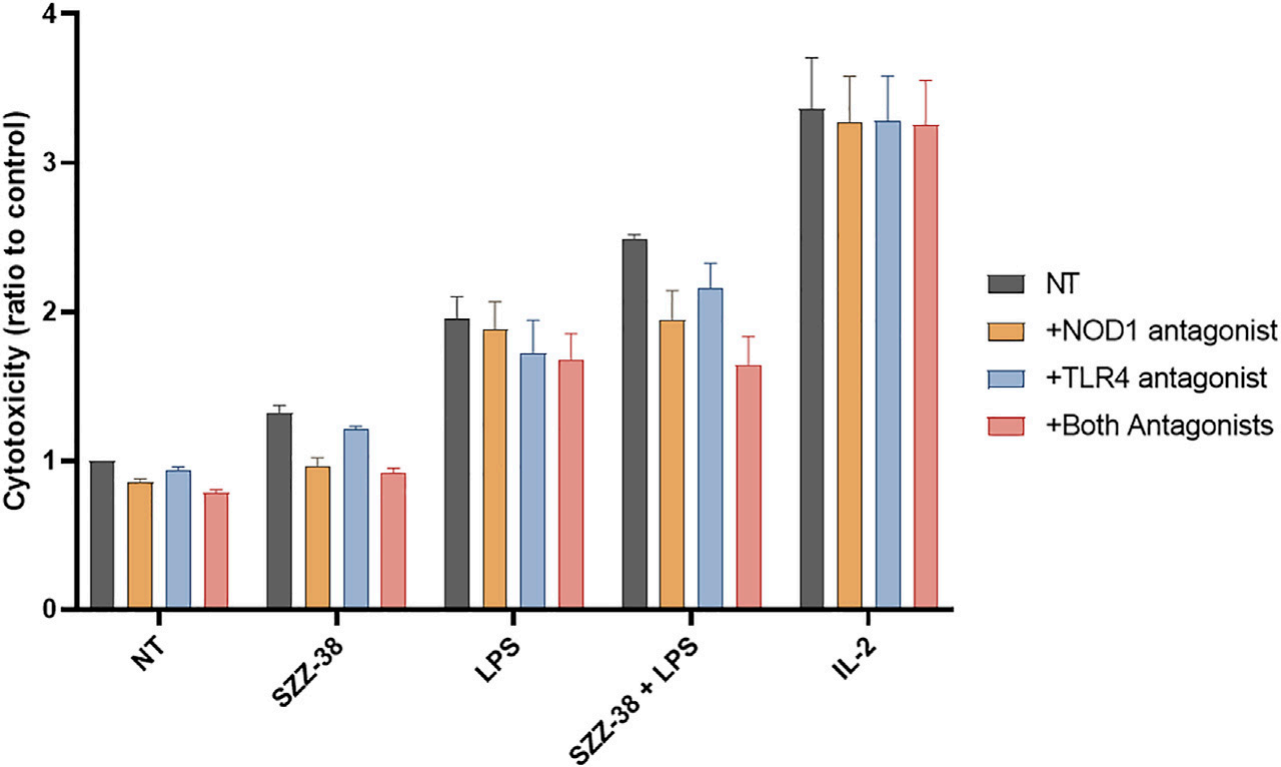
Effect of NOD1 and TLR4 antagonist pre-treatment on the SZZ-38- and LPS-induced PBMC cytolytic activity against K562 cells. PBMCs were pre-treated with the NOD1 antagonist Nodinitib-1 (10 µM), TLR4 antagonist TLR4-IN-C34 (100 µM), or both for 1 h, before the addition of SZZ-38 (1 µM), LPS (10 ng/mL), or IL-2 (200 IU/mL). Following 18 h activation, K562 cells were added. Cytotoxicity was determined after 4 h of co-incubation. Data are shown relative to the negative control (0.1 % DMSO) and expressed as means ± SEM of three independent experiments.

Next, the specificity of the observed synergistic effect on the cytotoxic capacity of PBMCs was examined. PBMCs were pre-treated with the previously reported NOD1 antagonist (Correa et al., 2011), TLR4 antagonist (Neal et al., 2013) or both antagonists prior to stimulation with SZZ-38 (1 µM), LPS (10 ng/mL), or a combination of SZZ-38 and LPS; IL-2 (200 IU/mL) was used as the positive control. The resulting cytolytic activities were evaluated and compared to those of the controls not being subjected to antagonist(s) pre-treatment. The relative reduction in cytolytic activities indicated that the effects of SZZ-38 and LPS are indeed a consequence of their NOD1 and TLR4 activation, respectively (Figure 5).

### Functional Analysis of Nucleotide-Binding Oligomerization Domain 1/Toll-Like Receptor 4-Promoted Natural Killer Cell Activation

**FIGURE 6:**
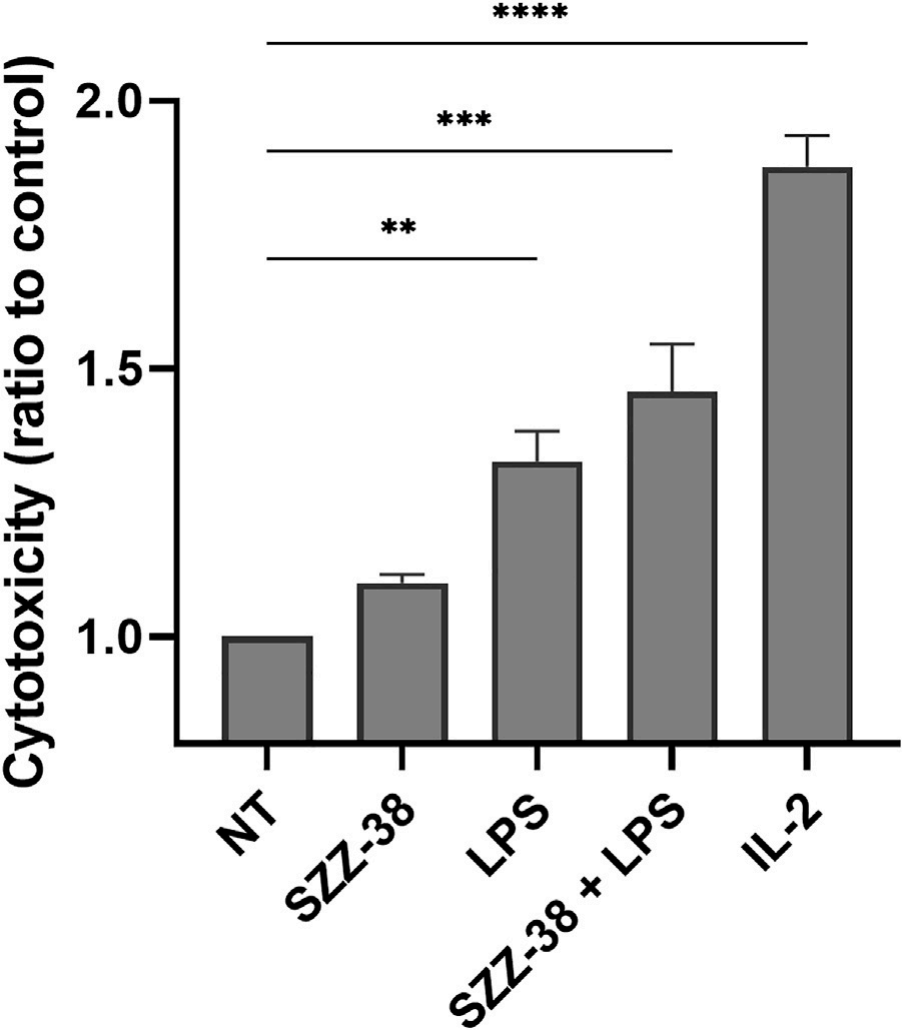
Effect of SZZ-38 and LPS on the cytolytic activity of NK cells against K562 cells. Isolated NK cells were treated for 18 h with SZZ-38 (1 µM), LPS (1 µg/mL), their combination, or IL-2 (200 IU/mL) before the addition of K562 cells. Cytotoxicity was determined after 4 h of co-incubation. Data are shown relative to the negative control (0.1% DMSO) and expressed as means ± SEM of three independent experiments. Statistical significance was determined by Dunnet’s multiple comparisons test; **, *p* < 0.01, ***, *p* < 0.001.

In a physiological system, the NOD1 agonist-engaged NK cell activity would most likely stem from the direct effects of NOD1 agonists on NK cells although an indirect activation of other NOD1-responsive accessory immune cells, including monocytes, *via* cytokine secretion cannot be excluded. For example, NK cell stimulation with iE-DAP has been shown to induce the secretion of tumor necrosis factor-α and IFN-γ (Qiu et al., 2010), while monocytes have been activated following NOD1 stimulation, alone and particularly in combination with IFN-γ (Sone et al., 1988; Inamura et al., 1989). To that end, we investigated whether NOD1 stimulation or a dual NOD1/TLR4 activation can augment the cytolytic activity of isolated NK cells. The experiment was conducted with NK cells isolated from whole blood by negative immunoselection; only cell preparations with a high content of CD3^-^/CD56^+^ cells were used in subsequent experiments. NK cells were then treated with NOD1 agonist SZZ-38 (1 µM) and LPS (1 µg/mL), alone or in combination, while IL-2 (200 IU/mL) served as the positive control; again, K562 were used as target cells, albeit at a 5:1 E/T cell ratio (Figure 6). Expectedly, SZZ-38 alone (1.10-fold) had negligible effect on the cytolytic activity of NK cells, LPS slightly enhanced this activity (1.33-fold) while IL-2 as the positive control considerably boosted the NK cell cytotoxicity against K562 (1.88-fold). The results obtained with isolated NK cells are comparable with those obtained in the PBMC-K562 assay in the sense that dual NOD1/TLR4 stimulation enhanced the NK cell cytotoxicity (1.47-fold). Granted, this increase in cytolytic capacity was additive and thus less pronounced than the synergistic increase observed when employing PBMCs as effector cells. It should however be noted that albeit non-synergistic in nature, the activation of NK cell subpopulation still can bring about a synergistic induction of the whole PBMC population that encompasses NK as well as accessory immune cells.

**FIGURE 7:**
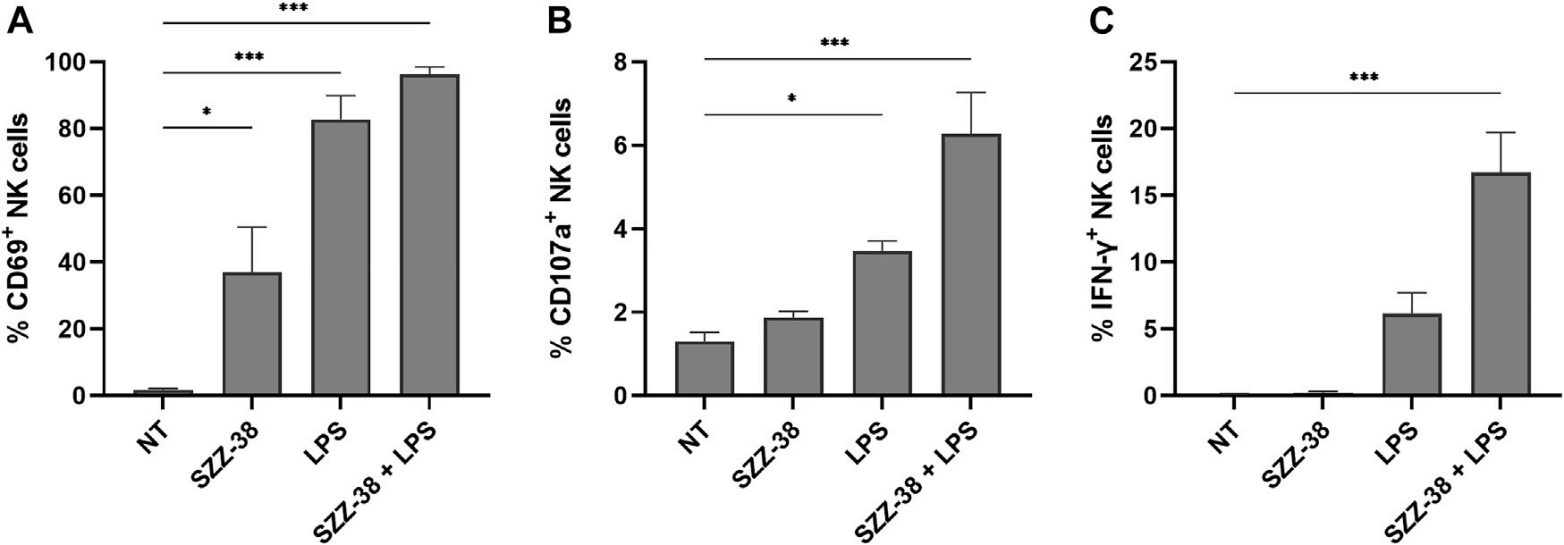
NK cell activation, degranulation, and IFN-γ production in response to NOD1 (SZZ-38, 1 µM) and TLR4 co-stimulation (LPS, 1 µg/mL). Live CD3^−^/CD56^+^ cells were gated as NK cells. Cell surface expression of CD69 **(A)** and CD107a **(B)**, and the percentage of IFN-γ producing **(C)** NK cells were assessed by cell surface and intracellular staining, followed by flow cytometric analysis. The results are expressed as percentages of CD69^+^ **(A)**, CD107a^+^ **(B)**, or IFN-γ^+^ **(C)** CD3^−^ CD56^+^ cells. The data represent the means ± SEM of three independent experiments. Statistical significance was determined by Dunnet’s multiple comparisons test; *, *p* < 0.05, ***, *p* < 0.001.

While assays, such as the PBMC/NK cell-K562 cytotoxicity assay, offer information about the end-stage effect, i.e., lysis of target cells, they provide no data on the level of activation of effector cell population. In order to provide an explanation for the observed synergistic effect of simultaneous NOD1/TLR4 activation on NK cell cytotoxicity, CD69 (early activation marker for NK cells), CD107a (degranulation marker) and IFN-γ (cytokine indicative of NK cell activation) expression levels were examined *via* multi-parameter flow cytometry by immunostaining of the CD3^-^/CD56^+^ subset of stimulated PBMCs (Figure 7). A strong relationship has namely been shown between these markers and target cell lysis (Khanna et al., 2021), with CD69 being one of the earliest cell surface activation markers expressed on stimulated NK cells (Dons’koi et al., 2011). SZZ-38 was able to activate NK cells by itself as evidenced by a significant upregulation of CD69, resulting in 37% of CD69^+^ NK cells, whereas surface expression of CD69 was expectedly low in unstimulated cells (2% of CD69^+^ NK cells). A similar, yet even more pronounced, increase was seen with LPS (83% of CD69^+^ NK cells), while a combined NOD1/TLR4 stimulation achieved a maximal stimulation and activated almost all NK cells, thus reaching frequencies of 96% CD69^+^ NK cells. Further analysis focused on CD107a expression as a functional marker of NK cell degranulation, which has been shown to correlate well with NK cell-mediated target cell lysis (Alter et al., 2004). The obtained results indicated that CD107a expression on the surface of NK cells was low in unstimulated cells (1.30%) and that stimulation with NOD1 agonist alone did not induce significant degranulation (1.88%). TLR4 stimulation, on the other hand, brought about an increase in degranulation (3.46%), while this effect was further enhanced by NOD1/TLR4 co-engagement (6.28%). NK cell functional activity was next quantitated by monitoring IFN-γ production *via* intracellular cytokine staining. Namely, in addition to their cytotoxic activity, NK cells are also among the most potent and early IFN-γ producers (e.g., during infection) (Selvanantham et al., 2013). As few as 0.09% of IFN-γ^+^ cells were detected in unstimulated cells. Similarly, NOD1 activation did little to increase the frequency of IFN-γ^+^ cells (0.33%). The TLR4 engagement by LPS, however, upregulated the levels of IFN-γ^+^ cells reaching frequencies of 6.15%. A combined NOD1/TLR4 stimulation showed a marked improvement as the proportion of IFN-γ^+^ cells was upregulated 185-fold versus the control, thus reaching frequencies of 16.73% of NK cells. Taken together, these data demonstrate that the frequencies of CD69^+^, CD107a^+^, and IFN-γ^+^ NK cells are significantly upregulated following NOD1/TLR4 co-engagement and the obtained results are in agreement with the observed cytolytic activities.

### Effect of Nucleotide-Binding Oligomerization Domain 1 Agonist on Interferon-γ-Induced Peripheral Blood Mononuclear Cells-Mediated K562 Cell Killing

**FIGURE 8:**
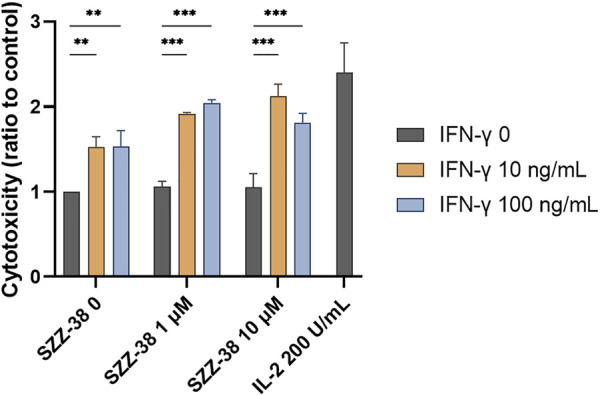
The concentration-dependent effect of NOD1 agonist/IFN-γ combinations on the induction of PBMC cytotoxicity against K562 cells. PBMCs were treated for 18 h with SZZ-38 (1 and 10 µM), IFN-γ (10 and 100 ng/mL), their combinations, or IL-2 (200 IU/mL) before the addition of K562 cells. Cytotoxicity was determined after 4 h of co-incubation. Data are shown relative to the negative control (0.1% DMSO) and expressed as means ± SEM of three independent experiments. Statistical significance was determined by two-way ANOVA followed by Dunnet’s multiple comparisons test; **, *p* < 0.01, ***, *p* < 0.001.

PBMCs promote the destruction cancer cells *via* direct cytolytic activity as well as indirectly by releasing cytokines, which in turn reinforce the recruitment and activation of other immune cells. We have shown that NOD1/TLR4 co-engagement increases the production of IFN-γ from the whole PBMC population as well as at the level of NK cells. Importantly, previous reports have also demonstrated the amplification effect of FK-565, a NOD1 agonist, on the IFN-γ-induced monocyte activation (Sone et al., 1988). This prompted us to test if SZZ-38 also shares this ability. We thus investigated whether a dual SZZ-38/IFN-γ activation can augment PBMC cytotoxicity. PBMCs were treated with different concentrations of SZZ-38 (at 1 and 10 µM) in combination with recombinant IFN-γ (at 10 and 100 ng/mL) (Figure 8). IL-2 as the positive control markedly boosted the PBMC cytotoxicity (2.40-fold). In comparison, maximum synergy was achieved by a combination of 10 µM SZZ-38 and 10 ng/mL of IFN-γ (2.12-fold). Similar results were obtained for a combination of 1 µM SZZ-38 and 100 ng/mL of IFN-γ (2.04-fold), while this level declined slightly in case of lower SZZ-38 concentration used (1 µM) (1.92-fold). Clearly, we have established the capacity of SZZ-38 to potentiate the IFN-γ-induced immune-cell mediated K562 cell killing.

## Conclusion

In summary, we shed more light on the structural requirements of iE-DAP derivatives for NOD1 agonism. In particular, the importance of assuming a correct orientation of the *meso*-DAP side-chain to fine-tune the NOD1 agonistic activity has been highlighted. Our most potent NOD1 agonist SZZ-38 acted cooperatively with LPS to amplify the release of both pro- and anti-inflammatory cytokines in PBMCs. Furthermore, SZZ-38 synergistically upregulated the LPS- as well as IFN-γ-induced PBMC-mediated K562 cell killing and optimal concentrations of all stimuli used have been identified. Combined NOD1/TLR4 stimulation also produced a similar, albeit additive, effect in isolated NK cells. A detailed mechanism of NK cell activation was provided, which revealed that the frequencies of CD69^+^, CD107a^+^, and IFN-γ^+^ NK cells are significantly upregulated following NOD1/TLR4 co-engagement. Collectively, our findings indicate a multifaceted role of NOD1 receptor in shaping the LPS-promoted non-specific immune response against K562 cancer cells. Presumably, a two-hit mechanism is at play here. Firstly, dual NOD1/TLR4 stimulation greatly contributes to PBMC/NK cell cytotoxicity and the production of IFN-γ and, importantly, NOD1 agonists can further enhance the IFN-γ responses. Our study thus attests to the importance of two-signal mechanisms, such as the demonstrated interplay between NOD1 and TLR4 pathways in boosting the non-specific immune response.

## Data Availability

The original contributions presented in the study are included in the article/Supplementary Material, further inquiries can be directed to the corresponding author.

## Abbreviations

BMDC, bone marrow-derived dendritic cell; C12-iE-DAP, lauroyl-γ-D-glutamyl-meso- diaminopimelic acid; DC, dendritic cell; E/T, effector to target; iE-DAP, γ-D-glutamyl-meso-diaminopimelic acid; IFN-γ, interferon-γ; IL, interleukin; LPS, lipopolysaccharide; MTS, 3-(4,5-dimethylthiazol-2-yl)-5-(3-carboxymethoxyphenyl)-2-(4-sulfophenyl)-2H-tetrazolium; NF-κB, nuclear factor κB; NK, natural killer; NLR, NOD-like receptor; NOD, nucleotide-binding oligomerization domain; PBMC, peripheral blood mononuclear cells; SEAP, secreted embryonic alkaline phosphatase; TLR, toll-like receptor.
