# Supplementary material for: Nucleotide-Binding Oligomerization Domain 1/Toll-Like Receptor 4 Co-Engagement Promotes Non-Specific Immune Response Against K562 Cancer Cells
Source: Front Pharmacol. 2022 Jul 22;13:920928. doi: 10.3389/fphar.2022.920928 (PMC9354050; doi:10.3389/fphar.2022.920928)
Supplement: Supplementary file 1 [file DataSheet1.docx]

Supplementary Material

# Supplementary Data

# Supplementary Figures and Tables

## Supplementary Figures


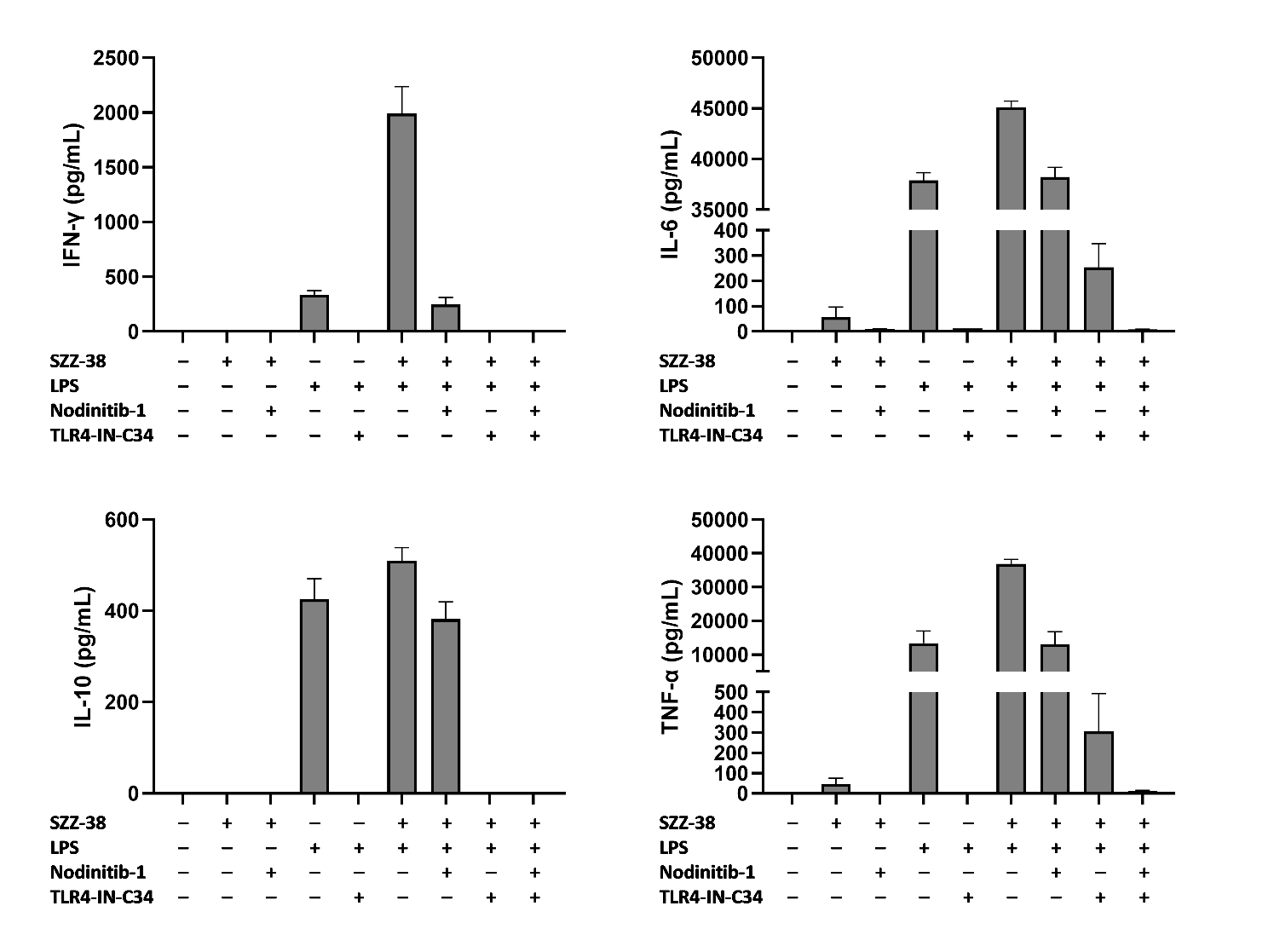


**Supplementary Figure 1.** Effect of NOD1 and TLR4 antagonist pre-treatment on the SZZ-38- and LPS-induced cytokine release from PBMCs. PBMCs were pre-treated with the NOD1 antagonist Nodinitib-1 (10 µM), TLR4 antagonist TLR4-IN-C34 (100 µM), or both for 1 h, before the addition of SZZ-38 (1 µM) and/or LPS (2 ng/mL). Corresponding vehicle was used as the negative control (0.1 % DMSO; NT). Following 18 h activation, cytokine release was measured. Data are expressed as means ± SEM of three independent experiments. Statistical significance was determined by one-way ANOVA followed by Bonferroni’s multiple comparisons test; ***, p < 0.001.
